# Spontaneous regression of extruded lumbar disc herniation: Correlation with clinical outcome

**DOI:** 10.12669/pjms.35.4.346

**Published:** 2019

**Authors:** Bilge Kesikburun, Emel Eksioglu, Aynur Turan, Emre Adiguzel, Serdar Kesikburun, Aytul Cakci

**Affiliations:** 1 Bilge Kesikburun, MD, University of Health Sciences, Dışkapı Yıldırım Beyazıt Training and Research Hospital, Department of Physical Medicine and Rehabilitation, Ankara, Turkey; 2 Emel Eksioglu, MD, Associate Professor, University of Health Sciences, Dışkapı Yıldırım Beyazıt Training and Research Hospital, Department of Physical Medicine and Rehabilitation, Ankara, Turkey; 3 Aynur Turan, MD, Associate Professor, Department of Radiology, University of Health Sciences, Dışkapı Yıldırım Beyazıt Training and Research Hospital, Department of Radiology, Ankara, Turkey; 4 Emre Adiguzel, MD, Associate Professor, University of Health Sciences, Gulhane School of Medicine, Department of Physical Medicine and Rehabilitation, Gaziler Physical Therapy and Rehabilitation Training and Research Hospital, Ankara, Turkey; 5 Serdar Kesikburun, MD, Associate Professor, University of Health Sciences, Gulhane School of Medicine, Department of Physical Medicine and Rehabilitation, Gaziler Physical Therapy and Rehabilitation Training and Research Hospital, Ankara, Turkey; 6 Aytul Cakci, MD, University of Health Sciences, Dışkapı Yıldırım Beyazıt Training and Research Hospital, Department of Physical Medicine and Rehabilitation, Ankara, Turkey

**Keywords:** Low back pain, Lumbar disc herniation, Pain, Spine, Spontaneous regression

## Abstract

**Objective:**

To evaluate the natural history of lumbar extruded disc with conservative treatment on MRI and to assess relation between the radiologic changes and clinical outcome.

**Methods:**

This prospective observational study was conducted at University of Health Sciences, Diskapi Yildirim Beyazit Training and Research Hospital between May 2015-June 2018. It included consecutive patients who were diagnosed as having lumbar symptomatic extruded disc as shown in MRI. After an average period of 17.0±7.2 months, repeat MRI was taken in 40 patients who received only conservative care during follow-up. Changes in the volume of herniated disc was measured. The patients were assigned into 3 groups as follow: (1) non-regression, (2) partial-regression, and (3) complete resolution. Numeric Rating Scale (NRS) pain score, the Oswestry Low Back Pain Disability Index (ODI) and muscle weakness were evaluated.

**Results:**

Based on disc volume of the T2-weighted MR images; four patients (10%) did not show any regression, six patients (15%) had a partial regression, and 30 patients (75%) had a complete resolution. Patients with complete resolution showed a significant improvement in the NRS pain score and the ODI score (p<0,001) over time. In patients with partial regression, only the ODI score improved significantly (p=0,043). Non-regression group did not show any improvement in any clinical outcome measure (p>0,05). Changes in the NRS scores over time were significantly higher in complete resolution group compared to non-regression group (p=0.016).

**Conclusion:**

The majority of the patients with extruded lumbar disc herniation might have reduction in size of herniated disc in the long run along with improvement in symptoms and function with conservative care.

## INTRODUCTION

Lumbar disc herniation is a common disorder that causes disability and poor quality of life by limiting functional capacity. Conservative approach or surgical interventions can be used in an individualized treatment plan based on clinical findings and patient preferences. The extruded lumbar disc herniation might generally be considered to be a schedule for surgical treatment, but most of the cases are suggested to be treated successfully by conservative treatment options.^1^

The natural history of disc herniation can be spontaneous regression over time as shown on imaging studies. In 1984, Guinto FC Jr et al.^2^ reported the first case of spontaneous regression of lumbar disc herniation. This condition has been described ranging 35%-100% for all grades of disc herniation in many studies.^3^ Lumbar disc herniation is enclosed by a granulation tissue comprising newly formed vessels and infiltrating macrophages. It is thought that the neovascularized zone with macrophage infiltration plays major role in the regression.^4^,^5^ Magnetic resonance imaging (MRI) is suggested as the best option for recognizing disc material and nerve roots. Disc morphology changes over time was shown using MRI in patients with lumbar disc herniation treated conservatively.^6^-^8^ MRI studies demonstrated that migrated and extruded disc herniation were more reduced in size.^9^,^10^

The spontaneous regression of herniated disc is a well-known issue. However, the correlation of morphologic changes in herniated disc with clinical outcome is not clear. The aim of this study was to evaluate the natural history of lumbar extruded disc with conservative treatment on MRI and to assess relation between the radiologic changes and clinical outcome.

## METHODS

A prospective observational study was undertaken at University of Health Sciences, Diskapi Yildirim Beyazit Training and Research Hospital between May 2015-June 2018. Patients with low back pain who were recruited from the outpatient clinic were examined to ascertain their eligibility. Sixty four consecutive patients (mean age 50.0±13.6 years) who were diagnosed as having lumbar symptomatic extruded disc as shown in MRI were included in the study. That levels with extruded hernia were consistent with symptoms and physical exam findings of the patients. The patients didn’t have another discopathy causing symptoms. Extruded disc herniation was defined as ‘in at least one plane, any one distance between the edges of the disc material beyond the disc space is greater than the distance between the edges of the base of the disc material’.^11^

### Exclusion Criteria

The exclusion criteria were:

1. Previous back surgery,
2. Cauda equina syndrome,
3. Low back pain combined with inflammatory diseases, vertebral fracture, spine infection and tumors
4. Central canal stenosis
5. Spondylolisthesis.

All participants provided written informed consent. The study adhered to the guidelines of the Declaration of Helsinki. The study protocol was approved by the Local Ethics Committee of Hospital.

Demographic data including age, gender, body mass index, duration of low back pain, and comorbidities were recorded. Severity of pain was assessed by numeric rating scale (NRS) ranging from 0 mm (no pain) to 10 mm (worst pain). The participants’ disability related low back pain was evaluated using a disease-specific functional status questionnaire, Oswestry Low Back Pain Disability Index (ODI). Each question of the ODI is rated on a scale from 0 to 5 points, with a higher score indicating high disability. The ODI scores range from 0 to 50. The Turkish validity and reliability of ODI was shown.^12^ Muscle weakness was implied as present or absent following a clinical examination at baseline. All patients were re-examined by the same specialist in rehabilitation medicine and re-imaged at follow-up visit. During follow-up period, patients received various conservative treatments including medication, physical therapy, exercise, acupuncture, and spinal injection.

### MRI

All patients were imaged using a 1.5T MRI scanner (Philips Achieva, Philips Medical Systems, Eindhoven, the Netherlands) with the spine coil in a supine position. Lumbar spinal MRI consisted of sagittal T1W, sagittal T2W, and axial T2W images. MRI was performed on all patients using the same MRI scanner at baseline and follow-up visit. MRI evaluation was carried out by the same experienced radiologist at baseline and follow-up. The radiologist was blind to the clinical information of the patients at any time point during the study. Volume of herniation (mm^3^) was calculated by measuring the area (mm^2^) in each axial image and multiplying this value by the scan thickness (4 mm) plus the inter-slice gap (1 mm).^13^ The patients were assigned into three groups based on change in extruded hernia disc volume as follow:

1. ***Non-regression:*** no changes in disc volume at all in sagittal and axial view
2. ***Partial-regression:*** markedly decreased herniated disc volume in sagittal and axial view
3. ***Complete resolution:*** disappeared herniated disc volume in sagittal and axial view.

### Statistical Analysis

Data analysis was performed using SPSS for Mac 20.0 software package program (SPSS Inc., Chicago, IL, United States). Data were presented as mean ± standard deviation [median (interquartile range)] for continuous variables and as frequencies and percentages for categorical variables. Conformity to normal distribution was assessed using the Shapiro-Wilk test. Comparison of the categorical variables was conducted with Chi-Square test. Comparisons within the groups were assessed using the Wilcoxon signed rank test. The Kruskal-Wallis test was used for comparisons between the groups. When statistically significant changes were found between the groups, two-up comparisons were conducted with the Bonferroni corrected Mann Whitney-U test and the results were considered to be statistically significant at p <0.017. In all other conditions, a value of p<0.05 was considered statistically significant.

## RESULTS

**Fig. 1:**
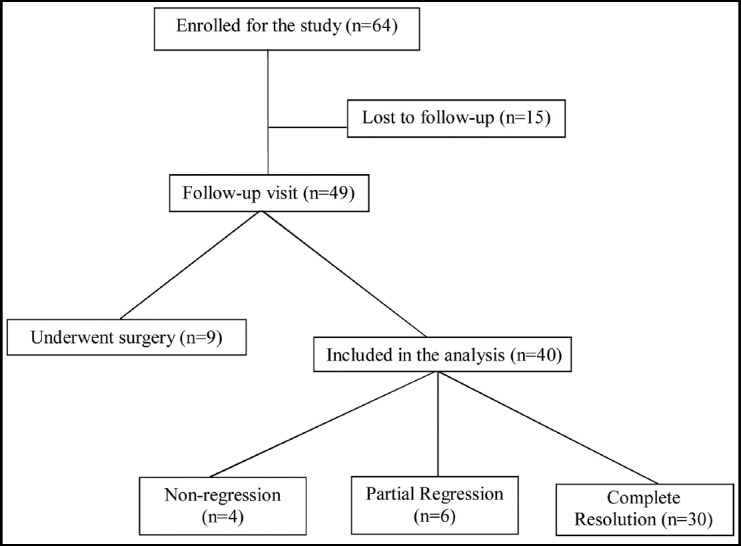
Flow chart.

Sixty four patients were enrolled for the study. Nine patients had lumbar disc surgery and 15 patients had lost to the follow-up visit. A total of 40 patients had a second MRI after the follow-up period, and so included in the statistical analysis (Fig.1). Mean follow-up time was 17.0±7.2 [14.0(12.0-19.0)] months. There was not significant difference in follow-up times between the groups (*p*=0.328). Based on disc volume of the T2-weighted MR images; four patients (10%) did not show any regression, six patients (15%) had a partial regression, and 30 patients (75%) had a complete resolution. In partial-regression group, volume of herniated disc reduced from 1433.1±558.5 [1397,5(855.0-2044.0)] mm^3^ to 528.5±331.5 [416,0(249.7-920.0)] mm^3^; the percentage of regression was 62.7±16.4 [54,6(50.3-82.3)] %. There was no significant difference in baseline demographic and clinical features of the patients between the groups (Table-I). Complete resolution group were inclined to be younger and to have less baseline volume of herniation compared to other groups, however they did not reach a statistically significant importance (*p*=0.119 and *p*=0.190, respectively).

**Table I:** Demographic and clinical features of the patients (n=40).

	Non-regression (n=4)	Partial Regression (n=6)	Complete Resolution (n=30)	p
Age (years)*	60.2±15.5 [65.5(43.7-71.5)]	54.6±14.0 [57.0(39.7-66.7)]	48.3±10.1 [50.5(41.7-57.0)]	0.119
Sex				0.732
*Female*	3 (75.0%)	3 (50.0%)	12 (40.0%)	
*Male*	1 (25.0%)	3 (50.0%)	18 (60.0%)	
BMI (kg/m2)*	29.3±4.2 [28.8(25.6-33.7)]	27.8±4.2 [28.2(23.9-31.9)]	28.7±5.6 [27.9(24.9-30.3)]	0.891
Marital Status				0.386
*Married*	4 (100.0%)	6 (100.0%)	25 (83.3%)	
*Single*	0 (0.0%)	0 (0.0%)	5 (16.7%)	
Occupation				0.449
*No*	4 (100.0%)	4 (66.7%)	22 (73.3%)	
*Yes*	0 (0.0%)	2 (33.3%)	8 (26.7%)	
Duration of Pain (months)*	6.2±0.9 [6.5(5.2-7.0)]	6.0±1.0 [6.0(5.5-7.0)]	5.9±1.8 [6.0(4.7-7.2)]	0.657
Level of Extruded Disc Herniations				0.471
*L1-L2*	0 (0.0%)	0 (0.0%)	1 (3.3%)	
*L2-L3*	0 (0.0%)	2 (33.3%)	2 (6.7%)	
*L3-L4*	0 (0.0%)	1 (16.7%)	2 (6.7%)	
*L4-L5*	3 (75.0%)	2 (33.3%)	12 (40.0%)	
*L5-S1*	1 (25.0%)	1 (16.7%)	13 (43.3%)	
Baseline Volume of Herniation (mm3)*	1367.0±828.8 [1420.0(567.0-2114.0)]	1433.1±558.5 [1397.5(855.0-2044.0)]	1045.6±736.2 [844.0(583.0-1327.5)]	0.190
Follow-up Time (months)*	21.5±7.5 [22.0(14.0-28.5)]	18.0±13.0 [13.5(10.5-23.7)]	16.2±6.3 [13.5(12.0-18.2)]	0.328

BMI: body mass index,

* Mean±SD [Median (Interquartile Range)].

**Fig. 2:**
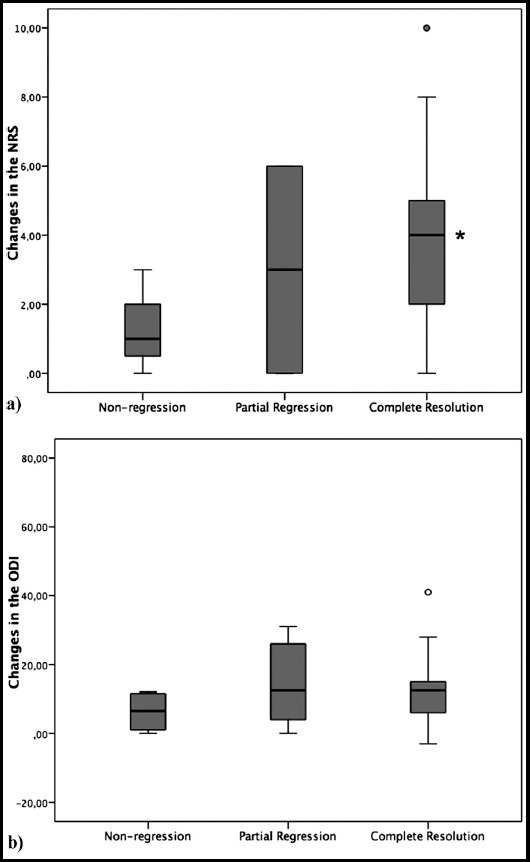
Comparison of changes in the NRS (a) and ODI (b) scores over time between the groups. ^*^Significant difference in complete resolution group compared to non-regression group (p<0.017, Mann-Whitney test).

The comparison of clinical outcome data between baseline and follow-up time points is shown in Table-II. Patients with complete resolution showed a significant improvement in the NRS pain score and the ODI score (*p*<0.001) over time. In patients with partial regression, only the ODI score improved significantly (*p*=0.043). Non-regression group did not show any improvement in any clinical outcome measure (*p>*0.05). Muscle weakness improved in three of five patients in complete resolution group and one patient in partial regression group. Improvement in muscle weakness did not reach a statistically significant change in any group (*p*=0.281). Changes in the NRS scores over time were significantly higher in complete resolution group compared to non-regression group (*p*=0.016) (Fig.2). Other two-up comparisons did not show significant differences in the changes in the NRS score and ODI score (p>0.05).

**Table II:** Clinical outcome data.

	n	Baseline	Follow-up	p
NRS*				
*Non-regression*	4	6.0±1.1[6.0(5.0-7.0)]	4.7±0.9[6.0(5.0-7.0)]	0.102
*Partial Regression*	6	6.6±1.2[6.5(5.7-8.0)]	3,6±2,2[2,5(2.0-6.2)]	0.063
*Complete Resolution*	30	6.9±1.7[7.0(5.7-8.0)]	2.9±2.0[3.0(1.0-4.2)]	<0.001
ODI*				
*Non-regression*	4	25.5±7.5[23.0(20.0-33.5)]	19.2±7.8[22.0(11.0-24.7)]	0.109
*Partial Regression*	6	28.6±10.3[29.5(18.7-38.5)]	14.3±9.9[12.0(6.2-22.7)]	0.043
*Complete Resolution*	30	23.3±11.8[20.0(14.0-32.2)]	12.1±9.3[11.0(4.0-18.5)]	<0.001
Muscle Weakness				0.281
*Non-regression*	4	0 (0.0%)	0 (0.0%)	
*Partial Regression*	6	1 (16.7%)	0 (0.0%)	
*Complete Resolution*	30	5 (16.7%)	2 (6.7%)	

NRS: Numeric Rating Scale, ODI: Oswestry Disability Index,

* Mean±SD [Median (Interquartile Range)].

## DISCUSSION

Current literature suggests lumbar disc herniation may regress spontaneously in a course of time without surgery. The present study inquired whether this chronic painful condition may relieve in the long run with disappearance of the herniated disc material. The findings revealed that three-fourths of the patients with extruded lumbar disc herniation had a complete resolution detected with a second MR imaging after an average follow-up period of 17 months. Those with complete resolution of extruded disc showed a significant pain relief compared to those with non-regressed disc herniation. In addition, low back pain disability improved in patients whose disc herniation regressed completely or partially.

Rate of spontaneous regression in patients with lumbar disc herniation varies depending on the study method, imaging modalities, inclusion criteria, follow-up time, morphologic classification of disc herniations and regression grading of herniations. Hong SJ et al.^14^ investigated the resorption rate of massive lumbar disc herniation in patients treated with transforaminal epidural steroid injection and reported that 85.7% (24/28) of patients had a partial or complete reduction in size of the herniation. Ahn et al.^6^ reported that that 67% of patients with extruded disc herniation had dramatic resolution after a mean of 4.3 months follow-up time. Benson et al.^15^ revealed that 83% (29/35) of the patients with massive lumbar disc herniation had a complete and sustained recovery at an average follow-up time of 23.2 months. Bush K et al.^8^ found that 64 of the 84 herniated/sequestered intervertebral discs (76.1%) showed a partial or complete resolution at one year on follow-up CT scanning. Komori H et al.^16^ reported that the patients with extruded disc herniation had 30.7% (8/26) partial regression and no complete resolution after a mean of 6.3 months follow-up time. Takada E et al.^17^ showed all 17 patients with extruded disc herniation (100%) had regression more than half in size with a two-year follow-up. It may be suggested that higher rate of regression and resolution for extruded lumbar disc herniation may be showed with a longer follow-up time. Chiu CC et al.^3^ reported that extruded and sequestrated type herniation were predictive factors for spontaneous regression of lumbar disc herniation and showed that the rate of extruded and sequestrated discs were 7.8 times higher than bulging or protruded discs. The present study included only the patients with extruded disc herniation. In addition, this study had relatively a longer follow-up time. So, the rate of spontaneous regression, especially complete resolution, might be found higher compared to the previous studies.

The relation between disc regression and clinical improvement has not been accounted for well. It was explained that not only compression of a nerve root by a herniated disc is responsible for the symptoms, but also the release of inflammatory cytokines and the composition of the herniated disc may contribute to the pain.^18^ Seo JY et al.^19^ reported that volumetric changes in herniated discs were not significantly correlated with clinical outcomes, while Hong SJ et al.^14^ showed that patients had significantly symptomatic improvements with disc regression. The present study found that pain and disability improved significantly with disc resorption.

Extruded disc herniation is generally an alerted condition for both clinicians and patients. Thus, surgery might be sometimes scheduled early due to pain without presence of absolute surgery indications such as progressive motor deficit and cauda equine. However, the present findings suggest that conservative management of the cases may lead to regression of extruded disc together with improved clinical outcome in the long run. Considering the likelihood of postoperative complications such as recurrence and failed back surgery syndrome^20^,^21^, conservative care might be prolongable for the patients whom refused early surgical treatment option, and meanwhile the patients might be monitored for a possible surgery decision with careful consideration of absolute indications. A recent prospective cohort study comparing surgical and conservative treatment for lumbar disc herniation suggested faster pain relief with surgery, but similar benefit with both treatments in the mid-term and long-term follow up.^22^ Atlas SJ et al.^23^ in their long-term lumbar spine study recommended individualized treatment plan required patients with lumbar disc herniation and their physicians to integrate clinical findings with patient preferences based on their symptoms and goals.

### Limitations of the study

The present study has achieved the goals that it aimed to substantiate. On the other hand, there are some limitations of this study. First, only the patients who chose not to undergo surgery were included in the study, introducing a potential bias in the population studied. Second, there was not a standardized time for second imaging, but it was longer than a year almost for everyone and it might be considered enough for showing long-term results. Third, even it was an observational cohort study, the rate of patients lost to follow-up is higher than expected. On the other hand, 40 patients were still enough to highlight the results of the study. Finally, patients underwent various conservative treatments during the follow-up period. So, the present results failed to show the impact of a specific conservative technique. Further prospective studies focused on a single conservative treatment with a control group might be performed to illuminate this issue.

## CONCLUSION

The majority of the patients with extruded lumbar disc herniation might have reduction in size of herniated disc along with an improvement in symptoms and function with conservative care. So prolonged conservative treatment might be taken into consideration for the patients who do not prefer early surgery in the absence of absolute neurogenic complications secondary to nerve compression.

### Author’s contribution

**KB**: Data collection and manuscript writing.

**EE**: Design the study.

**TA:** Analysis of data.

**KS:** Statistical analysis and critical review.

**AE**: Data collection.

**CA**: Critical review and final approval.
